# How has child maltreatment surveillance data been used in Canada?

**DOI:** 10.1186/1478-4505-12-65

**Published:** 2014-11-28

**Authors:** Lil Tonmyr, Wendy K Martin

**Affiliations:** Injury and Child Maltreatment Section, Public Health Agency of Canada, 785 Carling, AL 6807B, Ottawa, ON K1A 0K9 Canada

**Keywords:** Child maltreatment, Data collection, Knowledge uptake, Performance measurement, Research utilization, Surveillance

## Abstract

**Background:**

Recently, a survey was performed as part of a larger study at the Public Health Agency of Canada (PHAC) to develop and pilot a series of tools to measure the uptake and use of PHAC-produced or -supported knowledge products by its key partners and stakeholders. This article aims to i) examine the uptake and use of the *Canadian Incidence Study of Reported Child Abuse and Neglect 2008* (*CIS-2008*) and to ii) assess the utility of a knowledge uptake survey for collecting performance measurement data.

**Methods:**

Using the knowledge utilization ladder as a theoretical framework, a short survey was developed around the themes of reception, cognition, conversation, reference, effort, influence, and implementation. The survey was administered electronically to potential end-users of the CIS-2008. The final sample comprised 85 respondents.

**Results:**

The results demonstrated that the majority of the respondents were aware of CIS-2008 and had read and used it. A wide array of disciplines and sectors were identified as end-users. Types of use included discussion of CIS data with social workers, child welfare and health advocates, students, medical and legal professionals, and senior government decision makers. Further, CIS was referenced in reports, articles, policy research, community programs, and funding proposals and was used to influence or support the development of policies, programs, and projects. Valuable information on the use of surveillance reports, such as CIS-2008, can be gathered from a brief survey that was easy to administer, cost effective, and that respondents needed minimal time to complete.

**Conclusions:**

Piloting of the survey demonstrated that the tool, while not perfect, is quite useful for capturing performance measurement information; CIS-2008 is appreciated and used. There is an increased recognition of the importance of the CIS as a unique source of Canadian child maltreatment surveillance data that can influence and lead to the implementation of new programs and policies. Although suggestions for improvement of the CIS-2008 were provided, the present findings offer support for ongoing national child maltreatment surveillance.

## Background

The Public Health Agency of Canada (PHAC), along with its partners, conducts national child maltreatment surveillance through collection, analysis, interpretation, and dissemination of the *Canadian Incidence Study of Reported Child Abuse and Neglect* (CIS). To improve data quality and utility, with each cycle of the CIS, they solicit feedback from producers and users of surveillance information [1–5].

Surveillance data are a specific form of evidence designed to measure “disease” burden in person, time, and place [6]. The CIS may be understood as an evidence source between surveillance and research because it has information on risk indicators and short-term investigation outcomes. The CIS gathers data on five maltreatment types (exposure to intimate partner violence, neglect, emotional maltreatment, physical abuse, and sexual abuse), the extent of harm, the reporter of the maltreatment, short-term investigation outcomes, child and family circumstances, and child functioning issues [7]. Data are provided by child protection workers using definitions developed for the CIS. Child protection workers participated in a half-day training session and research assistants were able to respond to questions throughout the data collection period. They were told to use their clinical training and not legislation when they determined if maltreatment had occurred or not. Among five types of maltreatment, three could be captured on the form; each type could be substantiated, suspected, or confirmed. Mainstream and Aboriginal agencies collect data in all Canadian provinces and territories (P/Ts) to obtain national estimates of reported maltreatment and its severity in child protection agencies, augment understanding of the social determinants of health, investigate short-term investigation outcomes such as foster care, and monitor changes of substantiated maltreatment over time. To date, data have been collected in 1998, 2003, and 2008 using the same methodology [7–9].

Canada is a federal state where there are shared responsibilities for children’s safety, health, and wellbeing. P/Ts are responsible for child protection, while the Federal Government provides services on First Nations (Aboriginal) Reserves. As part of the federal government’s responsibilities for national surveillance, it is also required to monitor progress and demonstrate accountability and effectiveness of its programs, research, and science. It has several policies for its results-based management systems. In addition to regular evaluations and audits, the Treasury Board of Canada’s Management for Resources and Results Policy [10] requires ongoing collection and reporting of performance data annually and at various points throughout the fiscal year. Performance measurement information is intended to support ongoing program monitoring and improvements, as well as accountability reporting. To meet these obligations, the PHAC requires performance measurement methods and tools [11–13] that are relatively nimble and cost effective to allow for frequent, repeated data collection.

Since the late 1990’s, the child protection sector has increased its interest in understanding research uptake nationally and internationally [14–16]. However, less attention has been given to the uptake of surveillance data. After the release of CIS-2003, an evaluation was undertaken using a tool that addressed awareness of the CIS, if the CIS has influenced decision, implantation of policies and programs, and confirmation of local observations. Confirmation was addressed as instrumental (use research in a direct way to solve a problem), conceptual (use research indirectly in a general way), and symbolic (use research to justify decisions) utility [4]. From the findings, among senior decision makers at the local child protection agencies, we learned that the CIS data assisted with resource allocation, validation of local findings, and identification of trends [4]. Remote location, particularly apparent for Aboriginal agencies, may hamper engagement with the data through challenges in accessing researchers [4, 5]. Some respondents indicated that utility of the data were limited at the local agency level since many decisions were taken at the provincial ministry [4].

The present research is a part of a larger study undertaken at PHAC to adapt and pilot a variety of tools to measure the uptake and use of PHAC-produced or -supported knowledge products by its key partners and stakeholders, with the intent of identifying several tools that could be used for ongoing collection of performance measurement data [17–32]. Given that development and dissemination of surveillance knowledge products is a key function of PHAC, a survey adapted by de Goede et al., which operationalizes the “Knowledge Utilisation Ladder” was identified as a promising option, because it had been used to assess uptake and use of epidemiological knowledge products with policy makers and practitioners in the Netherlands [33–35].

The child maltreatment surveillance section at PHAC was interested in assisting with the tool development to assess the uptake of the CIS-2008 surveillance report with intended users. This research was deemed especially useful considering the abovementioned CIS-2003 evaluation using a different tool and sample [4, 5]. We anticipate that the present study will contribute to our ability to understand how the CIS-2008, and similar surveillance information, may be mobilized and utilized by partners to support further research and surveillance to inform policy development and to guide public health action. The objectives of the present analysis are to i) examine uptake and use of the CIS-2008 and ii) assess the utility of a knowledge uptake survey for collecting performance measurement data.

## Methods

### Sample

The sampling-frame comprised initially of a purposive sample of 170 individuals included in the child maltreatment surveillance dissemination database. The respondents were chosen since they or their organisation had been mailed the CIS report due to personal or organizational interest. The database included representatives from P/Ts, Federal Departments, educators, researchers, health practitioners and social workers, justice and law enforcement sector workers, and NGOs, including First Nations organisations. This database was updated to obtain accurate email addresses; however, we received 16 bounce-back emails and 7 autoreplies indicating absence during the study period. An additional 25 individuals self-identified as CIS users and requested to complete the survey which was granted. The enriched sample-frame (n = 195) provided a response rate of 38% (Cycle 1). Next, we conducted a second cycle of data collection. Cycle 2 comprised of two organizations involved with the health and welfare of children, namely the Canadian Paediatric Society, Child and Youth Maltreatment Section (membership unknown), and the Child Welfare League of Canada (membership of 140 organisations). Twelve surveys were completed; however, the response rate for Cycle 2 is unknown. The final sample comprised of 85 respondents.

### Data collection

We prepared a bilingual invitation letter outlining survey objectives. Cycle 1 was collected between February 28 and March 13, and Cycle 2 between March 8 and 22, 2013. Data were collected using a self-administered on-line survey, loaded on the FluidSurveys platform in English and French. For Cycle 1, two reminders were sent to the respondents. Cycle 2 was administered by the abovementioned organisations, which sent one reminder. The respondents averaged 6.25 minutes for completion. The survey was based on de Goede et al.’s adaptation of the ‘ladder of utilisation’ [33–35], developed to measure use of a national epidemiological report by government officials, public health professionals, and care providers in the Netherlands. Survey questions were slightly modified from earlier use by PHAC to include producers of the CIS report and add one open-ended question. Demographic information was also collected. We sought advice regarding ethical approval from PHAC’s research ethics board and were advised that it was not required. The respondents were assured confidentiality and the Survey platform de-identifies information; the abbreviated tool is shown below.

*Sample of questions asked regarding the use of the CIS-2008 (abbreviated version). Questions adapted from de Goede et al.* [33]:

1. I received the CIS-2008 report.
2. I have read and understood the information in the CIS-2008.
3. I have discussed the information in the Report with others (followed by examples).
4. I have cited the information in the Report in my work (policy briefs, emails, plans, reports, research, presentations, and other documents) (followed by examples).
5. I have tried to convey the importance of the information in the CIS-2008 Report to others in an effort to effect change (even if this was not successful) (followed by examples).
6. The information in the Report has influenced decisions, guidelines, policy, or program development (followed by examples).
7. The information in the Report has led to implementations of policies, programs, or guidelines (followed by examples).
8. Other than for the purpose of answering this survey, when did you last consult the CIS-2008?
9. Were you involved in the development of the Report as member of an advisory committee, reviewer, or contributor?
10. Do you have any comments about the CIS-2008 or about how you or others used the report (followed by examples)?

There are six stages in the “ladder of utilisation”, namely 1) Recognition – refers to the results of research being transmitted to the intended audience; 2) Cognition – indicates that the target audience have read and understood the results; 3) Conversation and Reference – signifies that the results have been referenced in the target audience’s written or oral communications; 4) Effort – denotes that effort was made to translate the results of the research by the target audience to their environment; 5) Influence – suggests that the research results inspired the target audience’s decisions; and 6) Implementation – means that the results provided purpose and direction to the target audience.

### Analysis

Results were summarised using FluidSurvey software to create descriptive statistics. We analysed the open-ended responses using Nvivo10 applying first-level qualitative content analysis techniques. Consequently, the responses were grouped by theme, namely reception, cognition, conversation, reference, effort, influence, and implementation [33, 34]. The responses pertaining to conversations were further analysed with respect to target audience (who?), format (how?), and information (what?). The responses regarding referencing the report were analysed regarding where and what aspect had been cited. The level of effort in conveying the importance of the report was analysed according to “who”, “how”, and “what”. The responses are presented in the order of the instrument.

## Results

### Participants

A quarter of the participants (n = 22) indicated that they had been involved as a member of a CIS advisory committee or as a reviewer or contributor. Producers of the CIS-2008 report were seen as important to include since they may be engaged in knowledge translation and as end-users of the CIS-2008. All Canadian jurisdictions were represented in the survey as well as international respondents. Almost half (47%, n = 35) worked in the research/education sector. About a quarter (27%, n = 20) worked for P/T and 20% (n = 15) for the federal government; 8% worked in the not-for-profit, 7% in private consulting, and 5% worked in the health care sector.

### Reception and cognition

Most of the respondents (75%, n = 64) indicated that they had received, read, and understood the CIS-2008. Among these respondents, the majority (n = 59, 90%) had also discussed the content with others. Many (80%, n = 52) had referenced the CIS-2008 in their work and 71% (n = 46) had conveyed the importance of the information to others; 56% (n = 36) reported that the CIS-2008 had influenced policies, programs, or guidelines and 39% (n = 24) indicated that it had resulted in their implementation. Approximately 75% of respondents (49/65) reported consulting the CIS-2008 at least once in the previous 6 months.

### Conversation

Overall, 90% of respondents had discussed the data with colleagues, government officials, researchers, students, and/or relatives. Others described the organizations and/or employment categories of the people including the level (i.e., local, P/T, federal, or international) with whom they had discussed the CIS-2008. Local organizations, such as child welfare agencies (including Aboriginal), domestic violence and child and family services, child welfare stakeholders (child welfare community advisory boards, foster parents), review panels, and hospital-based child abuse specialty teams, were mentioned. Others specified occupations including judges, police officers, child welfare workers, professionals in community health centres, child/youth mental health advocates, teachers, child care staff, clergy, and medical personnel. Respondents identified positions of people they had discussed the CIS-2008 findings with at the P/T level, including Cabinet Ministers, senior staff (Deputies, Assistant Deputy Ministers, Communication Directors, provincial children’s advocates), and policy analysts. The report was used to teach in colleges and universities: law (Children and the Law), social work (e.g., Aboriginal child welfare), and medical students. PhD students and dissertation supervisors engaged with the CIS-findings. The public was reached via media requests.

While the CIS-2008 was discussed informally, others presented findings formally through webinars and community forums. The report has also been the basis for written material such as government reports and fact and information sheets. One respondent summarised the report and led focus-group discussions to broaden understanding of the recommendations and findings.

The discussions focused on types of maltreatment and associated risk factors. Others used it to discuss prevention, children’s rights, service delivery, policy options in child welfare, and collaboration with partner organizations working on issues related to child maltreatment (e.g., caregiver alcohol and drug use). Some of these issues are detailed below.

### Reference

The information was cited by 80% (n = 52) of the participants in journal articles, book chapters, presentations, briefing notes, workshops, meetings, manuals, policy work, community programs, university courses, and funding proposals. When addressing the significance of the content referenced, several mentioned the need for reliable Canadian child maltreatment statistics.

“*Until the CIS, we had to rely largely on American statistics, and now we are able to understand more about child abuse and family violence from the Canadian perspective. It is particularly valuable to have the information sheets that are generated from this study. The long-term tracking of what is happening in Canada is invaluable from prevention, intervention, treatment and policy perspectives.*”

“*The CIS findings are critical to my ability to educate professionals and members of the general public. It is extremely important to have up-to-date, national data for Canada.*”

Respondents frequently cited maltreatment estimates from the report emphasising the importance of being able to compare provincial and national data. Others mentioned that the report provided needed data on specific populations such as Aboriginal children.

### Effort

In total, 71% had conveyed the importance of the CIS-2008 to effect change. Although many felt that they had already responded, they added detail, instead of the generic term “government” or government body, audiences were specified as cabinet minister, senate committee, or the United Nations Human Rights Committee.

Respondents repeated venues where the importance of the CIS-2008 was discussed; conferences, initiatives, or projects with their involvement. They also specified who was using the information from the report, e.g., child advocates. They conveyed the importance of child maltreatment surveillance data to:

-  Urge for prevention initiatives.
-  Address adult risk factors instead of focusing solely on children after harm occurred.
-  Develop a maltreatment questionnaire highlighting the importance of mandatory reporting.
-  Improve understanding between the intersections of child welfare and custody matters.
-  Raise awareness with service providers and governments.
-  Establish baseline to track changes over time.
-  Examine the impact of children witnessing violence.
-  Write a submission to propose changes to a provincial child welfare review.
-  Highlight the importance of child maltreatment research.
-  Set performance targets for programs.
-  Create cross-ministerial initiatives to prevent the need for child welfare.
-  Produce a knowledge transfer document that has been endorsed by almost 500 Canadian organizations.

### Influencing

Over half (56%) stated that the report had influenced decisions, guidelines, policy, or program development. The examples provided pertained to creating awareness and formulating a position regarding child maltreatment including:

-  The shift to differential response in child welfare.
-  The positioning of intimate partner violence and child maltreatment (reoccurring theme).
-  The education of Canadians about the situation in Canada.

The CIS-2008 influenced the development of educational materials for public legal education, general child abuse information, and a workshop on developmental trauma and public education campaign on the harm associated with physical punishment. The CIS-2008 also influenced the development of policies, new strategies, direction, and legislation. For instance, federally, the National Anti-Drug Strategy (childhood maltreatment and its link to youth substance abuse) and, provincially, the *Canadian Human Rights Tribunal on Equitable Funding for Child and Family Services Saskatchewan* and *Child Welfare Transformation*. The CIS assisted in developing differential response models in highlighting risk factors to inform different interventions to support families and reduce child welfare reliance. Some examples provided were unspecific, but the respondents were certain CIS was used as it was considered the definitive Canadian child protection data-source. Others stated that they regularly refer to CIS data when drafting papers and policies, including Aboriginal policy. Resource allocation and emphasis placed on an issue were also mentioned. Some respondents indicated expectations for future use, for example, analyses will be conducted to guide policy decisions regarding placement in Quebec.

In terms of influencing practice standards and guidelines, several topics were identified by the respondents, including child welfare and family law matters (child custody dispute), screening tools for family violence in child intervention, amendments to practice standards in British Columbia, and guidelines for agency surveys. The CIS-2008 influence on services was exemplified by a new neglect program, services for urban Aboriginal families, and the reengineering of intake. Finally, some responded that it was premature to establish the influence of CIS-2008.

### Implementation

Overall, 39% of respondents indicated that the CIS-2008 has led to the implementation of policies, programs, or guidelines. Some respondents provided general and specific examples, while others stated they had already answered this question earlier in the survey. General change was mentioned, such as an overall shift on how to address child maltreatment. For instance, less intrusive approaches to address socio-economic considerations for Aboriginal families, increased focus on intimate partner violence, differential response, corporal punishment challenge, and Aboriginal policy. Several respondents felt it was premature to expect implementation outcomes.

Some of the specific outcomes were aforementioned, others were new:

-  Decision to create a federal or P/T working group on family violence examining the intersection between the responses of the criminal, family, and child protection systems.
-  Aboriginal foster care criteria and life-plan framework is presently reviewed in Quebec.
-  Jordan’s Principle ^a^ working group.
-  An early joint referral project initiated with Legal Aid.
-  Early child development and family support policy development.
-  First Nations Human Rights Tribunal.
-  Differential response in Alberta and Ontario.

### Other comments

Some respondents highlighted the usefulness of CIS-2008 underlining its importance as a key document.

“*CIS-2008 is essential for my work and I wish that more cycles are forthcoming so we can better understand the evolution of rates of maltreatment, as well as the profile of children and families investigated by child protection in Canada.*”

Others described CIS-2008 as a good example of collaboration among governments, child welfare, and research sectors. They also saw it as a breakthrough for First Nations to become more involved in surveillance work. Many respondents emphasised that it is the only source of Canadian child maltreatment data underscoring that, prior to CIS-1998, no such data existed. The capturing of changes over time was also deemed essential. A few respondents highlighted the importance of collecting national-level data using a consistent tool. Many valued the rigour and clarity with which the CIS-2008 is written. Although stated positively, one respondent noted its methodological limitations.

Many respondents recommended that PHAC continue collecting, compiling, and providing these data for subsequent reports. Other suggestions addressed the scope of the CIS. They wanted an expansion to follow the child after intake and increased timeliness between collection and release of data.

## Discussion

The objectives of this study were to assess the uptake and use of CIS-2008 among the intended audience and examine the utility of the ladder of utilization for the collection of performance measurement data. Results demonstrate that the majority of the respondents were aware of the CIS-2008 and had read and used it. The richness of the examples of data use is very informative. A wide array of products, disciplines, and sectors were identified. Additionally, the survey was found to yield detailed performance information, while still being an easy to administer, cost effective tool, that did not create a significant response burden.

As evidenced by the responses, not all participants answered about CIS-2008 but about other cycles and knowledge products created from them. This may be seen as positive as PHAC had developed a dissemination strategy identifying multiple audiences and products in various formats [36]. The CIS investigation team also engaged decision makers in oral and written formats. In addition, since its inception, the CIS has had an advisory committee with representation of researchers and decision makers from across Canada. This approach was used to ensure its relevance across sectors and nationally.

These findings exemplify the use of the CIS and complement earlier findings [4, 5]. In the previous study, a minority used the data in decision-making, meanwhile over half of the current survey respondents demonstrated how the CIS had influenced policy and almost 40% found that the data had led to policy and program implementation. There may be several explanations for this finding: i) different target audiences – possibly the respondents in this survey are positioned to influence policy and program development and can, therefore, assess its utility – the previous survey suggested that respondents felt that provincial ministry personnel are better situated to influence policy than them; 2) different knowledge uptake tool – the authors hypothesised that the tool may have negatively influenced the results [5]; and 3) more data (three cycles) and subsequent analysis have been published (see a systematic review [37]). More time has elapsed since inception providing more time to influence and implement. Consistent with the literature, we recognise that decision-making is influenced by multiple sources [35].

Evidence-based practice is developed in an environment of increased accountability [38]. There were some indications that some respondents saw this survey as a vehicle to cut the CIS, which may have influenced participation and provided information. Ambiguity regarding the continuation of the CIS may also have influenced the results. PHAC did not plan for CIS-2013 due to an internal methodology review; this was not clearly communicated with partners. In the previous study, many suggestions were provided regarding how to disseminate and engage with users and how the content of the survey could be improved. In this survey, the general need for Canadian child maltreatment surveillance data was stressed. All research including the CIS, of course, suffers from biases across cycles, such as including only reported child maltreatment and methodological limitations like potential seasonal effects. To diminish some of these challenges, PHAC hopes to develop a three-pronged approach using population-based data in combination with administrative child welfare data and a targeted survey building on the CIS [39].

It is also noteworthy that surveillance data are only one type of evidence. Considering the dearth of Canadian child maltreatment data identified among several respondents, it is understandable that the expectations of the CIS are high and that some of these expectations fall outside the scope the CIS. This is consistent with the results of the CIS-2003 study [5] and further demonstrates that the limitations of surveillance data are unclear to many respondents. From a policy perspective, it is important to know the extent of child maltreatment in Canada. However, surveillance data cannot be expected to provide information for clinical practice interventions as a randomised controlled trial or systematic reviews. Instead, it must be utilized to provide evidence that then enables users to generate hypotheses for intervention research.

This study indicates that information from the CIS is accessed by an unusually broad group of users. This group ranges from members of the general public to students, professionals, and experts in such diverse disciplines as clinical medicine, public health, social work, law and justice, education, and sports and recreation, as well as faith groups. Among the health disciplines, public health has a broad interdisciplinary mandate and a commitment to improving health in every aspect of peoples’ lives; it is naturally linked to child maltreatment and associated risk factors and is well placed to engage in effective knowledge translation. Thus, CIS is a valuable resource to public health and PHAC.

The CIS-2008 provided an opportunity for capacity building. It was used by students for analysis and courses across disciplines. It also impacted professional development, for instance, by educating judges and lawyers. In terms of policy capacity it assisted in setting an agenda and informing the debate regarding the importance of child maltreatment. The results of this study demonstrated that the CIS can be used as an advocacy tool to garner attention to maltreatment and related concerns and allocating resources to priority areas and groups.

Several lessons were learned from this study. It demonstrated the importance of continuing to publish child maltreatment surveillance data. It underscored the need, in disseminating surveillance data, to clearly articulate the intended objective of the use. Furthermore, we obtained useful information from a brief survey that respondents needed minimal time to complete. In analysing the data, we felt that a wide range of examples were given but there was not sufficient information regarding the mechanism on how the data was used. For instance, we know that CIS data has been analysed regarding adolescent substance abuse [40] differences between Aboriginal and main stream agencies [41–43], but we do not know the direct influence of these analyses. There are two possible explanations, either the brief survey was not effective at capturing this information or there were challenges in understanding and measuring how data influence decision making. Furthermore, we do not know if all people working on a special program or policy would perceive that the decisions were influenced by the CIS. To address these concerns, later adaptations of the survey that were piloted used specific probes and examples to obtain more specific details [unpublished data, 2013].

At a practical level the use of partner organizations to disseminate the survey did not yield a good response rate. This finding is similar to the results from other PHAC surveys with other surveillance products [unpublished data, 2013]. Furthermore, the survey tool was at times deemed as repetitive. Thus, better operationalization of questions is warranted. Later PHAC pilots will be built on this lesson by merging several response categories together. Further, it terms of referencing the CIS, a citation analysis could have been undertaken to provide space for more in-depth responses to other questions. One drawback would be the challenge to capture the grey literature.

The instrument also implied a hierarchical structure which may not be useful for surveillance data. Surveillance data would need to be supplemented with other data. As pointed out in the literature, climbing the “ladder” implies considerable investment in skills to move to the next stage [34]. However, as noted by Lavis et al. [44], the meaning and significance of the uptake measure will differ depending on the context of the knowledge product. Not only may the use of knowledge vary between types of knowledge products, but different audiences may be expected to use the same knowledge in different manners. To ensure that the Knowledge Uptake survey measures knowledge uptake accurately, it is essential to be clear about what specific knowledge or information is being shared, how and to whom it is being shared, and what the anticipated type of use and ‘effect’ the knowledge is expected to have on the target audiences. To adapt the ladder of knowledge uptake model to suit the different types of knowledge products and anticipated types of use of PHAC’s knowledge products, it is important to assess the level of usage against the intended purpose or anticipated type of use, rather than always assessing usage based upon how far up the ladder usage occurs.

### Limitations and strengths

Our survey suffered from a low response rate, albeit acceptable for email-based surveys [45]; however, this may have influenced representativeness of responses. We experienced some electronic difficulties with the platform and it was challenging to keep up-to-date email lists. There was likely overlap regarding respondents between cycles and membership in the organizations.

Similarities of findings between this and the prior study provide validation of the results. Our sample size was sufficient to obtain a saturation of ideas. For instance, some open-ended questions, such as how the document has been referenced, could be rephrased into response categories. Some responses may suffer from desirability bias; however, it is probably less of an issue since examples of utilisation were provided.

## Conclusions

Piloting of the survey demonstrated that the tool, while not perfect, is quite useful for capturing performance measurement information. The survey elicits concrete examples of uptake and use, has a relatively low response burden, is easy to administer, and is cost effective – all of which are necessary for performance measurement tools that need to be administered in ongoing, yearly cycles.

The results of this study indicate that CIS-2008 is appreciated and used. There is an increased recognition of the importance of the CIS as a unique source of Canadian child maltreatment surveillance data that can influence and lead to the implementation of new programs and policies. Although suggestions for improvement of the CIS-2008 were provided, the study offers strong support for ongoing national child maltreatment surveillance.

## Endnote

^a^Jordan’s Principle is a child first principle named in memory of Jordan River Anderson. Jordan was a First Nations child from Norway House Cree Nation in Manitoba. Born with complex medical needs, Jordan spent more than two years in hospital while the Province of Manitoba and the Federal government negotiated over who should pay for his at-home care. Jordan died in hospital at the age of five. http://www.fncaringsociety.com/jordans-principle.

## Abbreviations

CIS: Canadian Incidence Study of Reported Child Abuse and Neglect
PHAC: Public Health Agency of Canada
P/Ts: Provinces and territories.
